# Supplementary material for: Pyruvate Dehydrogenase Kinase 1 inhibition mediated oxidative phosphorylation enhancement in cartilage promotes osteoarthritis progression
Source: BMC Musculoskelet Disord. 2023 Jul 20;24:597. doi: 10.1186/s12891-023-06585-6 (PMC10357736; doi:10.1186/s12891-023-06585-6)
Supplement: Supplementary file 1 — Additional file 1. [file 12891_2023_6585_MOESM1_ESM.zip › MF.Gsea.1653627316242/GOMF_TRANSCRIPTION_COREPRESSOR_ACTIVITY.html]

Details for gene set GOMF\_TRANSCRIPTION\_COREPRESSOR\_ACTIVITY[GSEA]

|  || Dataset | 55457\_collapsed\_to\_symbols.cls.cls #OA\_versus\_normal.cls.cls #OA\_versus\_normal\_repos |
| Phenotype | cls.cls#OA\_versus\_normal\_repos |
| Upregulated in class | normal |
| GeneSet | GOMF\_TRANSCRIPTION\_COREPRESSOR\_ACTIVITY |
| Enrichment Score (ES) | -0.3077619 |
| Normalized Enrichment Score (NES) | -1.4182914 |
| Nominal p-value | 0.013333334 |
| FDR q-value | 0.2584387 |
| FWER p-Value | 1.0 |
Table: GSEA Results Summary

  

Fig 1: Enrichment plot: GOMF\_TRANSCRIPTION\_COREPRESSOR\_ACTIVITY      
 Profile of the Running ES Score & Positions of GeneSet Members on the Rank Ordered List

  

| SYMBOL | TITLE | RANK IN GENE LIST | RANK METRIC SCORE | RUNNING ES | CORE ENRICHMENT || 1 | TSG101 | tumor susceptibility 101 [Source:HGNC Symbol;Acc:HGNC:15971] | 284 | 0.763 | -0.0065 | No |
| 2 | TLE6 | "TLE family member 6, subcortical maternal complex member [Source:HGNC Symbol;Acc:HGNC:30788]" | 385 | 0.711 | 0.0003 | No |
| 3 | DNMT3A | DNA methyltransferase 3 alpha [Source:HGNC Symbol;Acc:HGNC:2978] | 406 | 0.704 | 0.0133 | No |
| 4 | EID1 | EP300 interacting inhibitor of differentiation 1 [Source:HGNC Symbol;Acc:HGNC:1191] | 456 | 0.679 | 0.0235 | No |
| 5 | DHRS7B | dehydrogenase/reductase 7B [Source:HGNC Symbol;Acc:HGNC:24547] | 489 | 0.662 | 0.0346 | No |
| 6 | NR0B2 | nuclear receptor subfamily 0 group B member 2 [Source:HGNC Symbol;Acc:HGNC:7961] | 573 | 0.632 | 0.0412 | No |
| 7 | BRD7 | bromodomain containing 7 [Source:HGNC Symbol;Acc:HGNC:14310] | 726 | 0.588 | 0.0414 | No |
| 8 | HSBP1 | heat shock factor binding protein 1 [Source:HGNC Symbol;Acc:HGNC:5203] | 830 | 0.560 | 0.0449 | No |
| 9 | DPF2 | double PHD fingers 2 [Source:HGNC Symbol;Acc:HGNC:9964] | 854 | 0.554 | 0.0545 | No |
| 10 | TBL1XR1 | TBL1X receptor 1 [Source:HGNC Symbol;Acc:HGNC:29529] | 1070 | 0.501 | 0.0480 | No |
| 11 | SAP18 | Sin3A associated protein 18 [Source:HGNC Symbol;Acc:HGNC:10530] | 1099 | 0.496 | 0.0560 | No |
| 12 | URI1 | URI1 prefoldin like chaperone [Source:HGNC Symbol;Acc:HGNC:13236] | 1324 | 0.454 | 0.0478 | No |
| 13 | HDAC3 | histone deacetylase 3 [Source:HGNC Symbol;Acc:HGNC:4854] | 1325 | 0.454 | 0.0572 | No |
| 14 | CBX4 | chromobox 4 [Source:HGNC Symbol;Acc:HGNC:1554] | 1397 | 0.443 | 0.0608 | No |
| 15 | UXT | ubiquitously expressed prefoldin like chaperone [Source:HGNC Symbol;Acc:HGNC:12641] | 1641 | 0.407 | 0.0501 | No |
| 16 | CNOT7 | CCR4-NOT transcription complex subunit 7 [Source:HGNC Symbol;Acc:HGNC:14101] | 1676 | 0.402 | 0.0558 | No |
| 17 | C1D | C1D nuclear receptor corepressor [Source:HGNC Symbol;Acc:HGNC:29911] | 1848 | 0.379 | 0.0502 | No |
| 18 | PRKN | parkin RBR E3 ubiquitin protein ligase [Source:HGNC Symbol;Acc:HGNC:8607] | 1972 | 0.364 | 0.0481 | No |
| 19 | TAF9B | TATA-box binding protein associated factor 9b [Source:HGNC Symbol;Acc:HGNC:17306] | 2081 | 0.352 | 0.0469 | No |
| 20 | TCF25 | transcription factor 25 [Source:HGNC Symbol;Acc:HGNC:29181] | 2380 | 0.317 | 0.0300 | No |
| 21 | TRIM22 | tripartite motif containing 22 [Source:HGNC Symbol;Acc:HGNC:16379] | 2521 | 0.301 | 0.0253 | No |
| 22 | NCOR1 | nuclear receptor corepressor 1 [Source:HGNC Symbol;Acc:HGNC:7672] | 2549 | 0.297 | 0.0293 | No |
| 23 | RUVBL2 | RuvB like AAA ATPase 2 [Source:HGNC Symbol;Acc:HGNC:10475] | 2778 | 0.272 | 0.0170 | No |
| 24 | BCOR | BCL6 corepressor [Source:HGNC Symbol;Acc:HGNC:20893] | 2890 | 0.262 | 0.0137 | No |
| 25 | C1QBP | complement C1q binding protein [Source:HGNC Symbol;Acc:HGNC:1243] | 3083 | 0.241 | 0.0037 | No |
| 26 | SIAH2 | siah E3 ubiquitin protein ligase 2 [Source:HGNC Symbol;Acc:HGNC:10858] | 3147 | 0.235 | 0.0036 | No |
| 27 | CASP8AP2 | caspase 8 associated protein 2 [Source:HGNC Symbol;Acc:HGNC:1510] | 3186 | 0.232 | 0.0054 | No |
| 28 | RBFOX2 | RNA binding fox-1 homolog 2 [Source:HGNC Symbol;Acc:HGNC:9906] | 3523 | 0.200 | -0.0168 | No |
| 29 | TRIM28 | tripartite motif containing 28 [Source:HGNC Symbol;Acc:HGNC:16384] | 3526 | 0.200 | -0.0129 | No |
| 30 | EZH1 | enhancer of zeste 1 polycomb repressive complex 2 subunit [Source:HGNC Symbol;Acc:HGNC:3526] | 3565 | 0.197 | -0.0118 | No |
| 31 | RBBP8 | "RB binding protein 8, endonuclease [Source:HGNC Symbol;Acc:HGNC:9891]" | 3739 | 0.182 | -0.0216 | No |
| 32 | ZFPM2 | "zinc finger protein, FOG family member 2 [Source:HGNC Symbol;Acc:HGNC:16700]" | 3958 | 0.160 | -0.0354 | No |
| 33 | PFDN5 | prefoldin subunit 5 [Source:HGNC Symbol;Acc:HGNC:8869] | 3959 | 0.159 | -0.0321 | No |
| 34 | PEX14 | peroxisomal biogenesis factor 14 [Source:HGNC Symbol;Acc:HGNC:8856] | 3985 | 0.157 | -0.0308 | No |
| 35 | TDP2 | tyrosyl-DNA phosphodiesterase 2 [Source:HGNC Symbol;Acc:HGNC:17768] | 4124 | 0.144 | -0.0387 | No |
| 36 | FHL2 | four and a half LIM domains 2 [Source:HGNC Symbol;Acc:HGNC:3703] | 4127 | 0.143 | -0.0359 | No |
| 37 | DRAP1 | DR1 associated protein 1 [Source:HGNC Symbol;Acc:HGNC:3019] | 4462 | 0.116 | -0.0597 | No |
| 38 | COPS2 | COP9 signalosome subunit 2 [Source:HGNC Symbol;Acc:HGNC:30747] | 4494 | 0.113 | -0.0598 | No |
| 39 | SAP30 | Sin3A associated protein 30 [Source:HGNC Symbol;Acc:HGNC:10532] | 4907 | 0.079 | -0.0904 | No |
| 40 | BASP1 | brain abundant membrane attached signal protein 1 [Source:HGNC Symbol;Acc:HGNC:957] | 4923 | 0.078 | -0.0900 | No |
| 41 | CDYL | chromodomain Y like [Source:HGNC Symbol;Acc:HGNC:1811] | 5016 | 0.072 | -0.0957 | No |
| 42 | YAP1 | Yes1 associated transcriptional regulator [Source:HGNC Symbol;Acc:HGNC:16262] | 5041 | 0.070 | -0.0962 | No |
| 43 | CTBP2 | C-terminal binding protein 2 [Source:HGNC Symbol;Acc:HGNC:2495] | 5066 | 0.067 | -0.0967 | No |
| 44 | PHB | prohibitin [Source:HGNC Symbol;Acc:HGNC:8912] | 5102 | 0.064 | -0.0981 | No |
| 45 | NSD1 | nuclear receptor binding SET domain protein 1 [Source:HGNC Symbol;Acc:HGNC:14234] | 5178 | 0.058 | -0.1028 | No |
| 46 | HDAC9 | histone deacetylase 9 [Source:HGNC Symbol;Acc:HGNC:14065] | 5183 | 0.058 | -0.1019 | No |
| 47 | TFAP2A | transcription factor AP-2 alpha [Source:HGNC Symbol;Acc:HGNC:11742] | 5379 | 0.041 | -0.1163 | No |
| 48 | NPAT | "nuclear protein, coactivator of histone transcription [Source:HGNC Symbol;Acc:HGNC:7896]" | 5432 | 0.038 | -0.1196 | No |
| 49 | SNW1 | SNW domain containing 1 [Source:HGNC Symbol;Acc:HGNC:16696] | 5472 | 0.035 | -0.1220 | No |
| 50 | KDM5B | lysine demethylase 5B [Source:HGNC Symbol;Acc:HGNC:18039] | 5484 | 0.033 | -0.1221 | No |
| 51 | RUNX1T1 | RUNX1 partner transcriptional co-repressor 1 [Source:HGNC Symbol;Acc:HGNC:1535] | 5503 | 0.032 | -0.1229 | No |
| 52 | CBFA2T3 | CBFA2/RUNX1 partner transcriptional co-repressor 3 [Source:HGNC Symbol;Acc:HGNC:1537] | 5951 | -0.001 | -0.1579 | No |
| 53 | YAF2 | YY1 associated factor 2 [Source:HGNC Symbol;Acc:HGNC:17363] | 5995 | -0.004 | -0.1612 | No |
| 54 | RB1 | RB transcriptional corepressor 1 [Source:HGNC Symbol;Acc:HGNC:9884] | 6013 | -0.006 | -0.1624 | No |
| 55 | RCOR3 | REST corepressor 3 [Source:HGNC Symbol;Acc:HGNC:25594] | 6014 | -0.006 | -0.1623 | No |
| 56 | SCAI | suppressor of cancer cell invasion [Source:HGNC Symbol;Acc:HGNC:26709] | 6199 | -0.020 | -0.1763 | No |
| 57 | ANKRD1 | ankyrin repeat domain 1 [Source:HGNC Symbol;Acc:HGNC:15819] | 6416 | -0.037 | -0.1925 | No |
| 58 | PBXIP1 | PBX homeobox interacting protein 1 [Source:HGNC Symbol;Acc:HGNC:21199] | 6434 | -0.038 | -0.1930 | No |
| 59 | NCOR2 | nuclear receptor corepressor 2 [Source:HGNC Symbol;Acc:HGNC:7673] | 6497 | -0.042 | -0.1970 | No |
| 60 | EZH2 | enhancer of zeste 2 polycomb repressive complex 2 subunit [Source:HGNC Symbol;Acc:HGNC:3527] | 6562 | -0.047 | -0.2011 | No |
| 61 | SSX1 | SSX family member 1 [Source:HGNC Symbol;Acc:HGNC:11335] | 6693 | -0.055 | -0.2102 | No |
| 62 | HMGA2 | high mobility group AT-hook 2 [Source:HGNC Symbol;Acc:HGNC:5009] | 6850 | -0.067 | -0.2210 | No |
| 63 | ELANE | "elastase, neutrophil expressed [Source:HGNC Symbol;Acc:HGNC:3309]" | 6862 | -0.068 | -0.2205 | No |
| 64 | NR0B1 | nuclear receptor subfamily 0 group B member 1 [Source:HGNC Symbol;Acc:HGNC:7960] | 6885 | -0.070 | -0.2207 | No |
| 65 | HDGF | heparin binding growth factor [Source:HGNC Symbol;Acc:HGNC:4856] | 6924 | -0.074 | -0.2222 | No |
| 66 | FOXP3 | forkhead box P3 [Source:HGNC Symbol;Acc:HGNC:6106] | 7035 | -0.082 | -0.2291 | No |
| 67 | CITED2 | Cbp/p300 interacting transactivator with Glu/Asp rich carboxy-terminal domain 2 [Source:HGNC Symbol;Acc:HGNC:1987] | 7100 | -0.087 | -0.2323 | No |
| 68 | TLE2 | "TLE family member 2, transcriptional corepressor [Source:HGNC Symbol;Acc:HGNC:11838]" | 7198 | -0.096 | -0.2379 | No |
| 69 | LIMD1 | LIM domain containing 1 [Source:HGNC Symbol;Acc:HGNC:6612] | 7281 | -0.101 | -0.2423 | No |
| 70 | ATF7IP | activating transcription factor 7 interacting protein [Source:HGNC Symbol;Acc:HGNC:20092] | 7386 | -0.109 | -0.2482 | No |
| 71 | CCND1 | cyclin D1 [Source:HGNC Symbol;Acc:HGNC:1582] | 7512 | -0.119 | -0.2555 | No |
| 72 | WWTR1 | WW domain containing transcription regulator 1 [Source:HGNC Symbol;Acc:HGNC:24042] | 7824 | -0.143 | -0.2770 | No |
| 73 | GMNN | geminin DNA replication inhibitor [Source:HGNC Symbol;Acc:HGNC:17493] | 7866 | -0.146 | -0.2772 | No |
| 74 | HSPA1A | heat shock protein family A (Hsp70) member 1A [Source:HGNC Symbol;Acc:HGNC:5232] | 7876 | -0.146 | -0.2749 | No |
| 75 | TOB1 | "transducer of ERBB2, 1 [Source:HGNC Symbol;Acc:HGNC:11979]" | 7892 | -0.147 | -0.2730 | No |
| 76 | PRMT5 | protein arginine methyltransferase 5 [Source:HGNC Symbol;Acc:HGNC:10894] | 7904 | -0.148 | -0.2708 | No |
| 77 | HDAC1 | histone deacetylase 1 [Source:HGNC Symbol;Acc:HGNC:4852] | 7911 | -0.148 | -0.2682 | No |
| 78 | TLE4 | "TLE family member 4, transcriptional corepressor [Source:HGNC Symbol;Acc:HGNC:11840]" | 7915 | -0.149 | -0.2654 | No |
| 79 | GON4L | gon-4 like [Source:HGNC Symbol;Acc:HGNC:25973] | 7935 | -0.150 | -0.2638 | No |
| 80 | PPP1R13L | protein phosphatase 1 regulatory subunit 13 like [Source:HGNC Symbol;Acc:HGNC:18838] | 7967 | -0.153 | -0.2631 | No |
| 81 | CDY1 | chromodomain Y-linked 1 [Source:HGNC Symbol;Acc:HGNC:1809] | 8096 | -0.163 | -0.2697 | No |
| 82 | NRIP1 | nuclear receptor interacting protein 1 [Source:HGNC Symbol;Acc:HGNC:8001] | 8524 | -0.195 | -0.2992 | No |
| 83 | SIRT6 | sirtuin 6 [Source:HGNC Symbol;Acc:HGNC:14934] | 8532 | -0.196 | -0.2957 | No |
| 84 | MECP2 | methyl-CpG binding protein 2 [Source:HGNC Symbol;Acc:HGNC:6990] | 8687 | -0.208 | -0.3035 | Yes |
| 85 | SIRT7 | sirtuin 7 [Source:HGNC Symbol;Acc:HGNC:14935] | 8733 | -0.211 | -0.3026 | Yes |
| 86 | RERE | arginine-glutamic acid dipeptide repeats [Source:HGNC Symbol;Acc:HGNC:9965] | 8744 | -0.212 | -0.2990 | Yes |
| 87 | PAWR | pro-apoptotic WT1 regulator [Source:HGNC Symbol;Acc:HGNC:8614] | 8766 | -0.213 | -0.2963 | Yes |
| 88 | APEX1 | apurinic/apyrimidinic endodeoxyribonuclease 1 [Source:HGNC Symbol;Acc:HGNC:587] | 8767 | -0.213 | -0.2919 | Yes |
| 89 | CREG1 | cellular repressor of E1A stimulated genes 1 [Source:HGNC Symbol;Acc:HGNC:2351] | 8770 | -0.214 | -0.2876 | Yes |
| 90 | TLE1 | "TLE family member 1, transcriptional corepressor [Source:HGNC Symbol;Acc:HGNC:11837]" | 8895 | -0.224 | -0.2927 | Yes |
| 91 | DAXX | death domain associated protein [Source:HGNC Symbol;Acc:HGNC:2681] | 8926 | -0.226 | -0.2904 | Yes |
| 92 | SIRT1 | sirtuin 1 [Source:HGNC Symbol;Acc:HGNC:14929] | 8947 | -0.227 | -0.2873 | Yes |
| 93 | ZMYND11 | zinc finger MYND-type containing 11 [Source:HGNC Symbol;Acc:HGNC:16966] | 8952 | -0.228 | -0.2829 | Yes |
| 94 | RCOR1 | REST corepressor 1 [Source:HGNC Symbol;Acc:HGNC:17441] | 9120 | -0.241 | -0.2910 | Yes |
| 95 | TLE3 | "TLE family member 3, transcriptional corepressor [Source:HGNC Symbol;Acc:HGNC:11839]" | 9121 | -0.241 | -0.2860 | Yes |
| 96 | LCOR | ligand dependent nuclear receptor corepressor [Source:HGNC Symbol;Acc:HGNC:29503] | 9227 | -0.250 | -0.2891 | Yes |
| 97 | CBFA2T2 | CBFA2/RUNX1 partner transcriptional co-repressor 2 [Source:HGNC Symbol;Acc:HGNC:1536] | 9278 | -0.254 | -0.2877 | Yes |
| 98 | GPS2 | G protein pathway suppressor 2 [Source:HGNC Symbol;Acc:HGNC:4550] | 9338 | -0.258 | -0.2870 | Yes |
| 99 | DNAJB1 | DnaJ heat shock protein family (Hsp40) member B1 [Source:HGNC Symbol;Acc:HGNC:5270] | 9380 | -0.262 | -0.2848 | Yes |
| 100 | TBL1Y | transducin beta like 1 Y-linked [Source:HGNC Symbol;Acc:HGNC:18502] | 9416 | -0.266 | -0.2821 | Yes |
| 101 | LMCD1 | LIM and cysteine rich domains 1 [Source:HGNC Symbol;Acc:HGNC:6633] | 9464 | -0.270 | -0.2802 | Yes |
| 102 | ENO1 | enolase 1 [Source:HGNC Symbol;Acc:HGNC:3350] | 9606 | -0.284 | -0.2854 | Yes |
| 103 | CIR1 | "corepressor interacting with RBPJ, CIR1 [Source:HGNC Symbol;Acc:HGNC:24217]" | 9640 | -0.287 | -0.2821 | Yes |
| 104 | HNRNPU | heterogeneous nuclear ribonucleoprotein U [Source:HGNC Symbol;Acc:HGNC:5048] | 9847 | -0.306 | -0.2919 | Yes |
| 105 | SMARCA4 | "SWI/SNF related, matrix associated, actin dependent regulator of chromatin, subfamily a, member 4 [Source:HGNC Symbol;Acc:HGNC:11100]" | 10009 | -0.322 | -0.2979 | Yes |
| 106 | DNMT3B | DNA methyltransferase 3 beta [Source:HGNC Symbol;Acc:HGNC:2979] | 10089 | -0.330 | -0.2973 | Yes |
| 107 | SFMBT1 | Scm like with four mbt domains 1 [Source:HGNC Symbol;Acc:HGNC:20255] | 10094 | -0.330 | -0.2908 | Yes |
| 108 | CTBP1 | C-terminal binding protein 1 [Source:HGNC Symbol;Acc:HGNC:2494] | 10188 | -0.339 | -0.2910 | Yes |
| 109 | NAB2 | NGFI-A binding protein 2 [Source:HGNC Symbol;Acc:HGNC:7627] | 10196 | -0.340 | -0.2846 | Yes |
| 110 | TLE5 | "TLE family member 5, transcriptional modulator [Source:HGNC Symbol;Acc:HGNC:307]" | 10253 | -0.346 | -0.2818 | Yes |
| 111 | RYBP | RING1 and YY1 binding protein [Source:HGNC Symbol;Acc:HGNC:10480] | 10386 | -0.359 | -0.2847 | Yes |
| 112 | SRSF2 | serine and arginine rich splicing factor 2 [Source:HGNC Symbol;Acc:HGNC:10783] | 10507 | -0.374 | -0.2864 | Yes |
| 113 | PA2G4 | proliferation-associated 2G4 [Source:HGNC Symbol;Acc:HGNC:8550] | 10598 | -0.383 | -0.2856 | Yes |
| 114 | MTA2 | metastasis associated 1 family member 2 [Source:HGNC Symbol;Acc:HGNC:7411] | 10600 | -0.383 | -0.2777 | Yes |
| 115 | ZNF451 | zinc finger protein 451 [Source:HGNC Symbol;Acc:HGNC:21091] | 10601 | -0.383 | -0.2698 | Yes |
| 116 | TRIB3 | tribbles pseudokinase 3 [Source:HGNC Symbol;Acc:HGNC:16228] | 10839 | -0.409 | -0.2800 | Yes |
| 117 | CREBBP | CREB binding protein [Source:HGNC Symbol;Acc:HGNC:2348] | 10881 | -0.414 | -0.2746 | Yes |
| 118 | MED1 | mediator complex subunit 1 [Source:HGNC Symbol;Acc:HGNC:9234] | 11146 | -0.450 | -0.2860 | Yes |
| 119 | MYBBP1A | MYB binding protein 1a [Source:HGNC Symbol;Acc:HGNC:7546] | 11188 | -0.456 | -0.2798 | Yes |
| 120 | TBL1X | transducin beta like 1 X-linked [Source:HGNC Symbol;Acc:HGNC:11585] | 11263 | -0.466 | -0.2760 | Yes |
| 121 | N4BP2L2 | NEDD4 binding protein 2 like 2 [Source:HGNC Symbol;Acc:HGNC:26916] | 11271 | -0.467 | -0.2669 | Yes |
| 122 | MIER2 | MIER family member 2 [Source:HGNC Symbol;Acc:HGNC:29210] | 11375 | -0.483 | -0.2650 | Yes |
| 123 | PIAS1 | protein inhibitor of activated STAT 1 [Source:HGNC Symbol;Acc:HGNC:2752] | 11422 | -0.490 | -0.2585 | Yes |
| 124 | BCL3 | BCL3 transcription coactivator [Source:HGNC Symbol;Acc:HGNC:998] | 11431 | -0.493 | -0.2490 | Yes |
| 125 | RIOX2 | ribosomal oxygenase 2 [Source:HGNC Symbol;Acc:HGNC:19441] | 11444 | -0.495 | -0.2397 | Yes |
| 126 | SF1 | splicing factor 1 [Source:HGNC Symbol;Acc:HGNC:12950] | 11664 | -0.527 | -0.2460 | Yes |
| 127 | ATN1 | atrophin 1 [Source:HGNC Symbol;Acc:HGNC:3033] | 11682 | -0.529 | -0.2364 | Yes |
| 128 | TCP10L | t-complex 10 like [Source:HGNC Symbol;Acc:HGNC:11657] | 11731 | -0.538 | -0.2291 | Yes |
| 129 | MAP3K10 | mitogen-activated protein kinase kinase kinase 10 [Source:HGNC Symbol;Acc:HGNC:6849] | 11753 | -0.541 | -0.2196 | Yes |
| 130 | BTG2 | BTG anti-proliferation factor 2 [Source:HGNC Symbol;Acc:HGNC:1131] | 11917 | -0.576 | -0.2204 | Yes |
| 131 | TCERG1 | transcription elongation regulator 1 [Source:HGNC Symbol;Acc:HGNC:15630] | 11950 | -0.583 | -0.2109 | Yes |
| 132 | DDX54 | DEAD-box helicase 54 [Source:HGNC Symbol;Acc:HGNC:20084] | 11969 | -0.587 | -0.2002 | Yes |
| 133 | PIAS4 | protein inhibitor of activated STAT 4 [Source:HGNC Symbol;Acc:HGNC:17002] | 12060 | -0.610 | -0.1947 | Yes |
| 134 | SPEN | spen family transcriptional repressor [Source:HGNC Symbol;Acc:HGNC:17575] | 12108 | -0.622 | -0.1855 | Yes |
| 135 | HIRA | histone cell cycle regulator [Source:HGNC Symbol;Acc:HGNC:4916] | 12143 | -0.630 | -0.1752 | Yes |
| 136 | HMGB1 | high mobility group box 1 [Source:HGNC Symbol;Acc:HGNC:4983] | 12170 | -0.636 | -0.1641 | Yes |
| 137 | ZMYND8 | zinc finger MYND-type containing 8 [Source:HGNC Symbol;Acc:HGNC:9397] | 12214 | -0.649 | -0.1541 | Yes |
| 138 | NOC2L | NOC2 like nucleolar associated transcriptional repressor [Source:HGNC Symbol;Acc:HGNC:24517] | 12258 | -0.663 | -0.1437 | Yes |
| 139 | WNT4 | Wnt family member 4 [Source:HGNC Symbol;Acc:HGNC:12783] | 12331 | -0.686 | -0.1352 | Yes |
| 140 | IRF2BP1 | interferon regulatory factor 2 binding protein 1 [Source:HGNC Symbol;Acc:HGNC:21728] | 12387 | -0.704 | -0.1250 | Yes |
| 141 | ARID5A | AT-rich interaction domain 5A [Source:HGNC Symbol;Acc:HGNC:17361] | 12705 | -0.867 | -0.1320 | Yes |
| 142 | MTA1 | metastasis associated 1 [Source:HGNC Symbol;Acc:HGNC:7410] | 12792 | -0.952 | -0.1190 | Yes |
| 143 | AASS | aminoadipate-semialdehyde synthase [Source:HGNC Symbol;Acc:HGNC:17366] | 12799 | -0.968 | -0.0995 | Yes |
| 144 | CRYM | crystallin mu [Source:HGNC Symbol;Acc:HGNC:2418] | 12808 | -0.984 | -0.0798 | Yes |
| 145 | SIN3B | SIN3 transcription regulator family member B [Source:HGNC Symbol;Acc:HGNC:19354] | 12809 | -0.984 | -0.0595 | Yes |
| 146 | TOB2 | "transducer of ERBB2, 2 [Source:HGNC Symbol;Acc:HGNC:11980]" | 12838 | -1.041 | -0.0402 | Yes |
| 147 | HIPK2 | homeodomain interacting protein kinase 2 [Source:HGNC Symbol;Acc:HGNC:14402] | 12842 | -1.045 | -0.0189 | Yes |
| 148 | HDAC7 | histone deacetylase 7 [Source:HGNC Symbol;Acc:HGNC:14067] | 12870 | -1.131 | 0.0024 | Yes |
Table: GSEA details [plain text format]

  

Fig 2: GOMF\_TRANSCRIPTION\_COREPRESSOR\_ACTIVITY      
 Blue-Pink O' Gram in the Space of the Analyzed GeneSet

  

Fig 3: GOMF\_TRANSCRIPTION\_COREPRESSOR\_ACTIVITY: Random ES distribution      
 Gene set null distribution of ES for **GOMF\_TRANSCRIPTION\_COREPRESSOR\_ACTIVITY**
